# Osseoperceptive EMG Feedback Improves Grasp Force Control in Myoelectric Prosthetic Hands

**DOI:** 10.1002/advs.77462

**Published:** 2026-09-07

**Authors:** Cristian Felipe Blanco‐Diaz, Strahinja Dosen, Leonardo Cappello

**Affiliations:** ^1^ The BioRobotics Institute Scuola Superiore Sant'Anna Pisa Italy; ^2^ Department of Excellence in Robotics and AI The BioRobotics Institute Scuola Superiore Sant'Anna Pisa Italy; ^3^ Department of Health Science and Technology Aalborg University Aalborg Denmark

**Keywords:** artificial sensory feedback, closed‐loop control, grasp force regulation, myoelectric prostheses, osseoperception

## Abstract

Restoring sensory feedback is essential for achieving accurate and natural control of myoelectric prosthetic hands, yet most clinical systems remain open loop. Osseoperception, the transmission of vibrotactile and auditory sensations through bone‐conducted mechanical stimulation, has emerged as a promising approach for delivering artificial sensory feedback. In this study, osseoperceptive electromyography (EMG) feedback is used to convey the user's myoelectric signal through bone conduction, enabling predictive regulation of grasp force. A single bone transducer placed on the elbow provides three encoding schemes—vibrotactile, auditory, and combined vibrotactile–auditory—evaluated during a functional force‐matching task inspired by the Box and Block test. Thirteen non‐disabled participants and one transradial amputee performed grasp‐and‐transfer trials across five discrete force levels, with and without feedback. The main outcome measure was the success rate in producing the correct target force. In non‐disabled participants, auditory‐only encoding significantly improved force‐control accuracy compared to no feedback (71% vs. 50%), whereas vibrotactile‐only and combined encoding showed positive but non‐significant trends (both 60%). The amputee participant achieved the best performance with vibrotactile encoding. These findings demonstrate the feasibility of osseoperceptive EMG feedback for predictive grasp‐force regulation using a single lightweight actuator and support its potential as a non‐invasive strategy for closed‐loop prosthetic control.

## Graphical abstract

Osseoperception is explored as a non‐invasive pathway for delivering sensory feedback in myoelectric prosthetic control. By encoding the user's electromyographic (EMG) signal through bone‐conducted stimulation, this approach enables predictive grasp force regulation. Results show improved control accuracy and user perception using a single actuator, highlighting its potential for simplified, multimodal closed‐loop prosthetic systems.

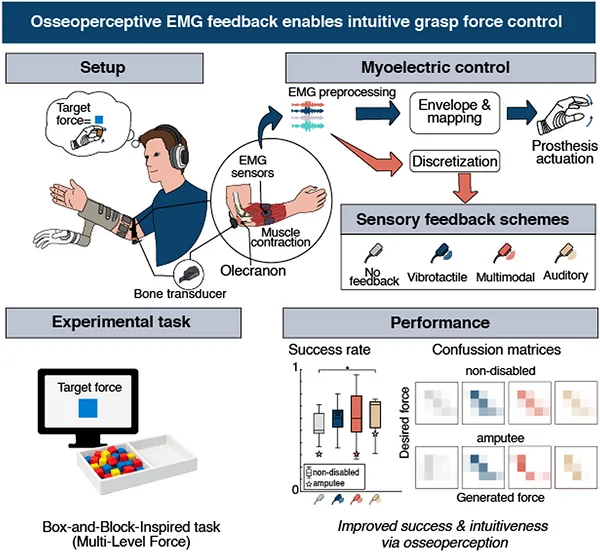

## 1 Introduction

Upper‐limb myoelectric prostheses have enabled individuals with amputation to recover basic motor functions, such as grasping and object manipulation [1, 2]. However, restoration of motor output alone is insufficient to achieve natural and effective hand function, as most commercially available prostheses do not provide somatosensory feedback to the user [3]. Numerous studies have shown that sensory feedback is essential to establish and maintain stable and accurate motor behavior [4]. Consistently, high abandonment rates and user dissatisfaction with myoelectric prostheses have been attributed, at least in part, to the lack of explicit somatosensory feedback from the device.

In the intact sensorimotor system, grasping relies on the continuous integration of afferent sensory signals generated during contact between the digits and the object. These signals are used to time and modulate feedforward motor commands, enabling precise and coordinated actions [5]. In prosthesis users, the absence of cutaneous feedback often forces reliance on visual or incidental auditory cues, which are delayed, cognitively demanding, and less intuitive [3, 6]. As a result, interaction with the environment and manipulation of objects can become unreliable.

Artificial sensory feedback can be used to convey information about the outcomes of prosthesis actions with the purpose of restoring the sensorimotor control loop [7]. Such feedback can be modality‐matched, when the artificial stimulus resembles the natural sensory modality which it replaces (e.g., tactile stimuli for touch events), or modality‐mismatched, when information is conveyed through an alternative sensory channel (e.g., auditory cues encoding tactile interactions) [8]. Grasp force is the information most commonly fed back to literature study participants [9, 10]. This is not surprising, as reliable force control when grasping an object is crucially important; applying excessive force could potentially damage the object, whereas the object could be dropped if the grasp is not strong enough.

A wide range of invasive and non‐invasive feedback strategies have been explored, including direct nerve stimulation, electrotactile feedback, mechanotactile interfaces, and vibrotactile stimulation of the skin [11, 12, 13, 14, 15]. These approaches have been shown to improve grasp force regulation as well as facilitate the adaptation of feedforward motor commands across trials [16]. In particular, vibrotactile sensory substitution has been extensively studied, with force magnitude encoded by tuning the amplitude or spatial location of the vibrotactile stimuli delivered to the forearm [3, 16, 17]. Although these approaches can improve prosthesis control, many feedback systems still require a substantial available skin area or complex hardware configurations, which may limit their practical integration in individuals with different amputation levels and residual limb characteristics [15, 18].

Recently, the concept of Electromyography (EMG) feedback has been introduced as an alternative strategy, in which the amplitude of the myoelectric signal driving the prosthesis is conveyed back to the user through artificial haptic stimulation [3, 17, 19]. Since prostheses are commonly controlled proportionally to contraction intensity, closing velocity and grasp force are coupled. By continuously informing the user about the current level of muscle activation during prosthesis closing, EMG feedback allows the user to adjust contraction intensity before object contact occurs, thereby supporting predictive force control strategies [17, 20]. Although promising results have been reported when delivering vibrotactile EMG feedback, they rely on multiple vibromotors distributed around the forearm. This poses a practical limitation for daily use in users with short stumps or articular amputations, while also increasing hardware complexity due to the need for multiple actuators, wiring, calibration procedures, and available mounting space [15, 17, 19].

Bone‐conducted stimulation offers a promising and underexplored alternative for delivering supplementary sensory feedback. Mechanical vibrations applied to bone can elicit localized vibrotactile sensations—via mechanoreceptors in the skin and periosteum—or auditory sensations when the mechanical waves reach the cochlea, a phenomenon known as osseoperception [21, 22]. Such a dual sensory pathway enables the transmission of rich sensory information using a single, lightweight actuator. This can be exploited to replace multiple conventional vibromotors, leading to compact devices, an important factor for clinical applications. Prior studies have demonstrated that stimulation frequency and intensity can modulate the perceived sensory modality. However, most existing research on osseoperception has focused primarily on psychophysical assessments [21, 23, 24]. In our earlier work, we demonstrated that multimodal osseoperceptive feedback can improve grip‐force control and motor coordination during pick‐and‐lift tasks [22]. In that study, osseoperceptive feedback encoded touch events and grasp force generated after object contact and the task was based on avoiding object breakage above a predefined force threshold. However, manipulation during activities of daily living extends beyond fragile‐object interactions. Many everyday objects differ in properties such as compliance, stiffness, or weight, requiring the user to continuously regulate grasp force across multiple levels [3, 15].

Therefore, in this study we introduce osseoperceptive EMG feedback for closed‐loop control of a robotic hand prosthesis. Instead of conveying contact force feedback, we transduce the user's own myoelectric signal into bone‐conducted stimulation. We evaluated three encoding schemes—vibrotactile‐only, auditory‐only, and mixed vibrotactile–auditory—using a single bone transducer placed on the elbow to accommodate both non‐disabled participants and transradial amputees. We aimed at investigating if the multimodal nature of bone conduction can be exploited to provide EMG feedback and enable predictive force control using a single stimulator device. Fourteen participants, including one transradial amputee, performed functional force‐matching tasks inspired by the Box and Block test, with five discrete target force levels spanning the full range of prosthesis force, under feedback and no‐feedback conditions.

We hypothesized that osseoperceptive EMG feedback—conveying the user's own myoelectric control signal rather than the resulting grasp force—would improve force control accuracy compared to no feedback, and that different encoding schemes would exhibit distinct trade‐offs between accuracy and subjective workload. However, we also expected that osseoperceptive feedback would likely decrease the speed of task execution due to additional corrections that can be performed when the feedback is available [3].

## 2 Material and Methods

### 2.1 Participants

Fourteen non‐disabled participants (age range: 22–35 years; 9 males and 5 females) and one female participant with a left transradial amputation (55 years old, with prior experience using a myoelectric prosthesis; amputation occurred 10 years prior to the study) were recruited for this study. All participants were right‐hand dominant, except for the participant with limb loss. The study was approved by the Ethics Committee of the North Denmark Region (Approval No. N‐20250021), and written informed consent was obtained from all participants.

### 2.2 Experimental Setup

The experimental setup consisted of four main components: (1) a multifunctional myoelectric prosthetic hand (Michelangelo, Ottobock, GE); (2) a surface electromyography (EMG) system used for prosthesis control; (3) an artificial sensory feedback interface based on bone‐conduction stimulation to elicit osseoperception; and (4) the standard equipment for the “Box and Block” test. The end‐to‐end latency was not measured experimentally. Nevertheless, based on manufacturer datasheets and separate component‐level estimates for the EMG acquisition hardware, signal processing, Bluetooth communication, prosthesis controller, and bone‐conduction transducer, the total system latency is estimated to be approximately 120 ms.

The sensory feedback interface featured a bone‐conduction transducer (B‐81, Radioear, US), which was positioned over the olecranon (OL) of the elbow to mechanically stimulate the underlying bone and induce vibrotactile sensations via osseoperception [23, 24]. The prosthetic hand and the sensory feedback unit were interfaced with a personal computer (PC) via Bluetooth and USB connections, respectively. System control, signal processing, feedback generation, and the graphical user interface were implemented in Python (v3.10.13) and executed on a 64‐bit operating system (Intel Core i7‐13620H, 16 GB RAM). Visual instructions and task cues were presented to participants on an 18‐inch computer monitor placed on the table, approx. 50 cm away from the participant.

To minimize external auditory interference and suppress airborne sound produced by the prosthetic motors, participants wore high‐attenuation foam earplugs (35.5 dB; Tokelu, Germany) in combination with active noise‐canceling headphones (Peltor X5A, 3 M, USA) throughout each experimental session. This configuration was adopted to restrict sensory feedback predominantly to bone‐conducted stimulation and to create an acoustically isolated environment for sensory assessment [21, 23, 24]. Importantly, this will not be necessary in the practical application since the stimulator would be, in that case, integrated inside the socket, providing sound insulation.

#### 2.2.1 Hand Prosthesis

**FIGURE 1:**
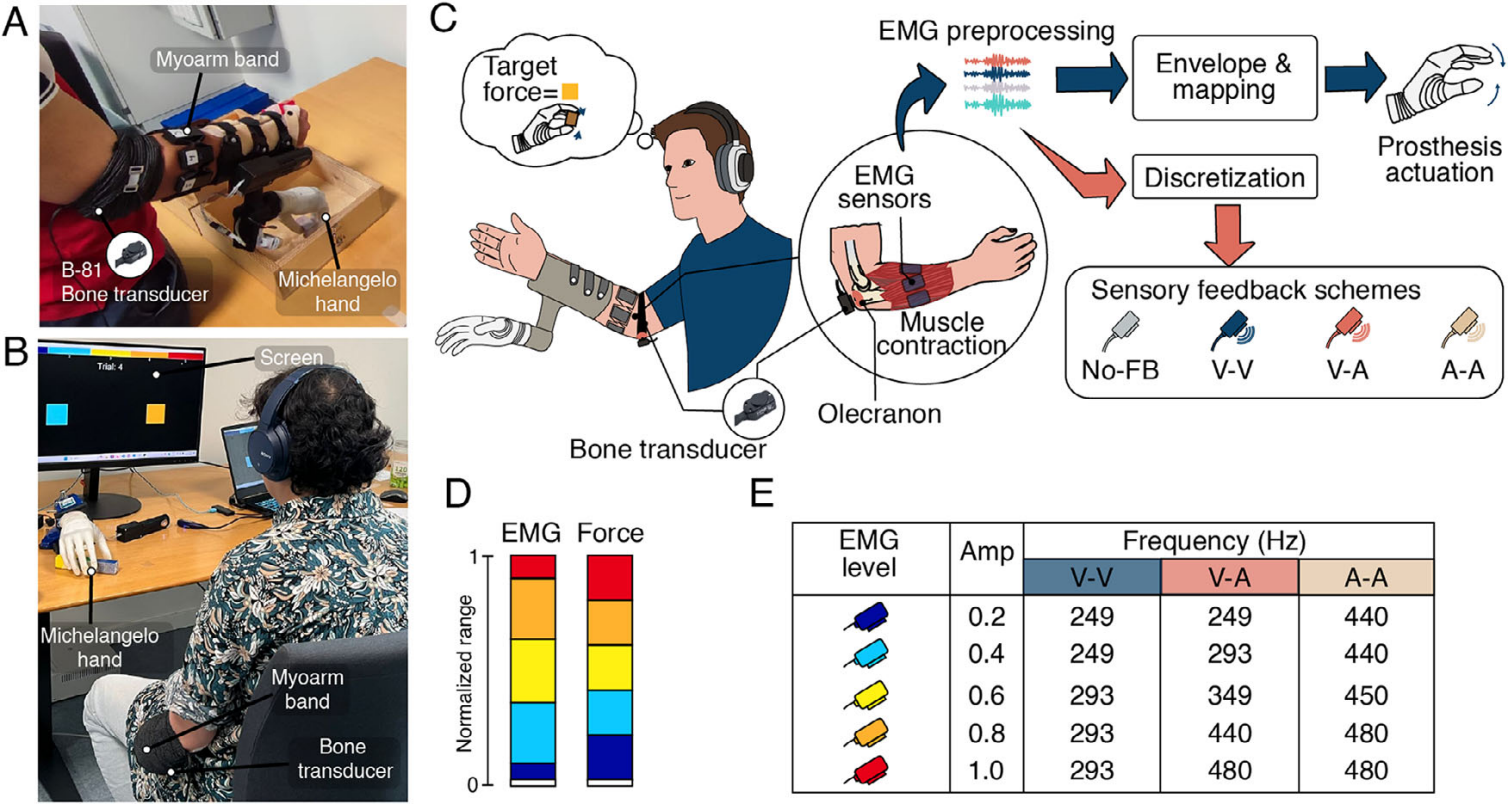
Experimental setup and closed‐loop myoelectric control with osseoperceptive feedback. (A) Experimental configuration for non‐disabled participants, showing the attachment of the Michelangelo prosthetic hand, the placement of the EMG interface on the forearm, and the bone‐conduction transducer mounted over the olecranon for sensory feedback delivery. (B) Configuration for the participant with transradial amputation, in which the prosthetic hand was rigidly mounted on a table fixture due to the absence of a compatible socket. (C) Schematic of the closed‐loop myoelectric control strategy, in which wrist flexor activation‐controlled prosthesis closing velocity and grasping force. (D) During prosthesis closing, discrete levels of the myoelectric signal were encoded as artificial sensory feedback, enabling predictive regulation of the grasping force prior to object contact (E) Sensory feedback parameters were implemented using the bone‐conduction transducer, including vibrotactile‐only (V–V), auditory‐only (A–A), and combined vibrotactile–auditory (V–A) modalities, each comprising five discrete stimulation levels. The amplitude (amp) is expressed as normalized to the maximum intensity (1).

For the able‐bodied participants, a custom 3D‐printed mount was used to attach the prosthetic hand to the distal forearm (Figure 1A). To minimize wrist motion and enforce isometric forearm muscle contractions during myoelectric control, the participants’ wrists were immobilized using a thermoplastic splint (ORLIMAN). This configuration was adopted to reduce confounding effects due to wrist movements and to ensure consistent muscle activation patterns across trials [3].

Due to the contingent lack of a compatible socket interface for the participant with left transradial amputation, a customized experimental configuration was implemented (Figure 1B). In this condition, the prosthetic hand was rigidly mounted on a bench vice, which maintained a fixed orientation and ensured mechanical stability throughout the experimental tasks. The setup also provided a clear view on the prosthesis, hence allowing the participant to exploit full visual feedback of prosthesis motion (similarly as during the actual use).

The Michelangelo prosthetic hand (Ottobock, Germany) is a non‐backdrivable, multifunctional myoelectric prosthesis with mechanically coupled long fingers capable of opening and closing, as well as an actuated thumb that can move into opposition [3]. This design allows the execution of multiple grasp types, including lateral and pinch grasps, and can be combined with an active wrist rotation unit. In the present study, lateral grasp and wrist rotation functionalities were disabled, restricting control to opening and closing movements in pinch grasp mode only.

The prosthesis is equipped with an embedded force sensor that measures the grasping force generated between the thumb and index finger. According to manufacturer specifications, the maximum force output in pinch mode is approximately 70 N [3].

#### 2.2.2 Myoelectric Control

Myoelectric control was implemented using a commercial wearable EMG interface (Myo Armband, Thalmic Labs, Canada), which provides eight evenly spaced surface electrodes positioned circumferentially around the proximal forearm. The armband was placed over the wrist flexor and extensor muscle groups, which were identified for each participant through palpation during voluntary contractions.

EMG signals were sampled at 200 Hz, high‐pass filtered using a second‐order Butterworth filter with a cutoff frequency of 20 Hz to remove motion artifacts, and notch‐filtered at 50 Hz to suppress power line interference [22]. The preprocessed signals were transmitted to a personal computer via Bluetooth for real‐time processing.

A calibration procedure was performed at the beginning of each session to acquire training data for three muscle activation conditions: rest, wrist flexion, and wrist extension. Participants were instructed via a visual interface to maintain each condition for 5 s, repeated over four consecutive cycles. Based on the calibration data, EMG channels corresponding predominantly to wrist flexor and extensor activity were automatically identified and assigned to prosthesis closing and opening commands, respectively [3, 17]. Activation of wrist flexor muscles resulted in prosthesis closing, whereas activation of wrist extensor muscles resulted in prosthesis opening (Figure 1C).

For control signal generation, the selected EMG channels were rectified and low‐pass filtered with a cutoff frequency of 3 Hz to extract a smooth envelope suitable for proportional control [3, 17]. To reduce fatigue and ensure consistent control across participants, the envelope amplitude was normalized to 60% of the maximal voluntary contraction (MVC). A dead zone threshold of 10% MVC was applied to prevent involuntary prosthesis activation due to unintentional low‐level muscle activity.

The normalized myoelectric signal was divided into five discrete activation levels and mapped to the prosthesis command input using a piecewise linear mapping function (Figure 1D), as commonly adopted in proportional myoelectric control schemes [3, 17, 20]. The resulting command signal served as a reference input to the prosthesis motor controller. When the prosthesis was not in contact with an object, the command input regulated the closing velocity. Upon contact with a rigid object and subsequent motor stalling, the same command input determined the grasping force level. As the prosthetic hand is non‐backdrivable, the force achieved at contact was maintained until an opening command was issued via wrist extension.

#### 2.2.3 Sensory Feedback Interface

The sensory feedback interface was implemented using a bone‐conduction transducer (B‐81, Radioear, US; dimensions: 4.5 × 3.0 × 1.0 cm; weight: 20 g), positioned over the OL of the elbow. The transducer was mounted using a custom 3D‐printed support and secured with adjustable elastic bands and Velcro straps to ensure stable coupling throughout the experiment [22, 24]. The flat surface of the transducer was placed in direct contact with the skin overlying the target bone, and a static preload force of approximately 3–5 N was applied to promote efficient bone conduction while minimizing vibration propagation to adjacent soft tissues [24].

The transducer was driven by an ESP32 microcontroller (Espressif Systems, CN), which generated sinusoidal waveforms through a digital‐to‐analog converter (DAC). The output signal was updated at 5 ms intervals and synchronized with prosthesis state updates. Signal frequency and amplitude were programmable within the operational bandwidth of the transducer (0.1–10 kHz). The waveform was amplified using an LM386 audio amplifier (Texas Instruments, USA) to ensure compatibility with standard audio transducers.

Prior to experimental sessions, the output of the bone‐conduction transducer was calibrated using an artificial mastoid (Model 4930, Brüel & Kjær, DK) to verify amplitude and frequency accuracy across the stimulation range [24]. Based on this calibration, stimulation parameters were selected to maximize the use of the available dynamic range while remaining within perceptually comfortable limits for each participant.

Previous studies have demonstrated that modulation of stimulation amplitude and frequency delivered via bone‐conduction transducers can evoke controlled vibrotactile sensations, auditory sensations, or a combination of both [24]. Based on this literature, three feedback conditions were implemented: vibrotactile‐only (V–V), auditory‐only (A–A), and combined vibrotactile–auditory (V–A). In each condition, the bone‐conduction transducer was used to provide EMG feedback encoding the continuous prosthesis control signal into five discrete levels (see Figure 1E for stimulation parameters). Therefore, the sensory feedback provided discrete information related to the myoelectric control signal during prosthesis closing (Figure 1C). Each of the five myoelectric activation levels—corresponding to distinct grasping force levels that would be generated after contact—was mapped to a unique stimulation pattern characterized by specific amplitude and/or frequency settings of the bone‐conduction transducer (Figure 1D,E). As the myoelectric signal increased from lower to higher intervals, the stimulation intensity increased accordingly. The discrete encoding scheme was selected due to its robustness, ease of interpretation, and prior successful application in vibrotactile prosthetic sensory feedback systems [3, 17].

During prosthesis closing, the sensory feedback enabled participants to monitor their level of muscle activation and thereby predict the grasping force that would be generated upon object contact. Participants were instructed to use the feedback to reach and maintain a target myoelectric activation level until full hand closure. As the prosthesis is non‐backdrivable, the grasping force corresponding to the myoelectric level at contact was maintained after closure. While participants could increase force by generating higher muscle activation following contact, controlled decreases in force were not possible without opening the hand, consistent with the operational characteristics of commercial myoelectric prostheses.

Before commencing the experimental tasks, a verification procedure was conducted for each participant. Stimulation parameters were adjusted to ensure that the intended sensory modality was reliably perceived in each condition (i.e., vibrotactile sensation in V–V, auditory sensation in A–A, and combined perception in V–A). Participants were required to correctly discriminate and identify all five discrete stimulation levels within each feedback condition before proceeding to the experimental tasks.

### 2.3 Experimental Protocol

Each experimental session consisted of five conditions: an initial no‐feedback condition (NoFB1), followed by three osseoperceptive feedback conditions—vibrotactile‐only (V–V), auditory‐only (A–A), and combined vibrotactile–auditory (V–A)—and a final no‐feedback condition (NoFB2) (Figure 1E). The order of the three feedback conditions was pseudo‐randomized across participants. The inclusion of no‐feedback conditions before and after the feedback blocks was intended to assess both the immediate effects of artificial sensory feedback and potential carryover or training effects on myoelectric control performance [16].

Each condition comprised three consecutive phases: (1) calibration and familiarization, (2) training, and (3) evaluation. At the beginning of each condition, EMG calibration was performed as described in Section 2.2.2. Participants then underwent a familiarization phase to learn the association between myoelectric activation levels and the corresponding sensory feedback patterns. During this phase, prosthesis motion was disabled. Participants generated wrist flexion contractions to reach each of the five predefined myoelectric levels, while receiving the corresponding sensory feedback, i.e., stimulation at a frequency and amplitude specified in Figure 1E, depending on the tested condition. Simultaneously, a colored square representing the active level was displayed on the screen to provide explicit visual confirmation (Figure 1D,E).

Following familiarization, participants completed the training phase, during which active prosthesis control was enabled, and the experimental task was performed. The task was inspired by the Box and Block test [3] and required participants to use the prosthetic hand to grasp a cubic object with the desired force and to transport it over a central barrier from one side of the workspace to the other. A trial was considered successful when the object was lifted and placed on the opposite side of the barrier while the grasping force was maintained at the desired level. During training, two visual indicators were displayed on the screen: the right square indicated the target force level to be achieved, while the left square provided real‐time visual feedback corresponding to the current prosthesis force level. Participants were instructed that this visual feedback represented the grasping force of the prosthesis, whereas the artificial sensory feedback conveyed information about their myoelectric activation level. If, as explained before, the participants adjusted their muscle contraction to the desired myoelectric level, the prosthesis would close and achieve the desired force level; hence, the task was successfully executed. After closing the hand, the participants could also relax their muscles since the prosthesis was non‐backdrivable and would maintain the achieved force until an explicit opening command was given. For each force level, participants performed five trials, resulting in a total of 25 pseudorandomized trials per condition.

After completing the training phase, participants proceeded to the evaluation phase. This phase followed the same task structure but differed in two key aspects. First, the minimum and maximum force levels were excluded from the set of desired forces, as these levels were considered trivial to achieve across conditions and did not allow meaningful assessment of sensory feedback effectiveness [3, 17]. Second, real‐time visual feedback was removed by deactivating the left square on the PC screen, requiring participants to rely solely on the artificial sensory feedback and their prior task familiarization. In this phase, participants performed 15 trials per remaining three force levels, for a total of 45 trials per condition.

In the no‐feedback conditions (NoFB1 and NoFB2), the sensory feedback interface was deactivated. In all conditions, participants accessed to incidental visual information and natural muscle proprioception to approximate realistic prosthesis use scenarios.

Due to the absence of a prosthetic socket, the experimental protocol for the participant with transradial amputation was adapted. Specifically, the task was restricted to generating and maintaining the target grasp force without object transport, as stable prosthesis attachment was not available. Under this adapted protocol, a trial was considered successful when the participant reached the prescribed force level and maintained it within the corresponding interval for at least 2 s. Once this criterion was met, the trial was terminated, and the object was released. To reduce muscle fatigue and ensure reliable EMG acquisition, the number of evaluation trials was reduced to 10 trials per force level, resulting in 30 trials per condition for this participant.

### 2.4 Data Analysis

The primary outcome measure used to assess force control accuracy was the success rate, defined as the percentage of successful trials. A trial was considered successful when the participant correctly grasped and transferred the block while achieving the instructed grasping force level. Given the discrete feedback encoding adopted in this study, a successful trial required that the force level generated by the prosthesis—defined as the maximum force level attained during the trial—matched the target force level.

The secondary outcome measure assessed task execution speed and was defined as the time required to complete a successful trial measured from the trial start to the moment the participant opened the hand and dropped the object. Success rate was computed both overall and separately for each target force level and participant, whereas task completion time was computed per force level and condition.

To further evaluate participants’ ability to generate distinct force levels under different feedback modalities, confusion matrices were constructed using pooled results across participants. Each matrix compares the instructed (desired) force level against the achieved (generated) force level, allowing assessment of systematic errors in force generation and the intuitiveness of the sensory feedback encoding.

Subjective perception of the different feedback modalities was assessed using the NASA Task Load Index (NASA‐TLX) questionnaire to quantify perceived workload. In addition, participants were asked to rank the sensory feedback conditions according to comfort, intuitiveness, perceived integration, and invasiveness. These subjective measures were analyzed descriptively.

Statistical analyses were conducted using data from non‐disabled participants only and were performed in MATLAB R2022a (MathWorks, USA). One non‐disabled participant was excluded from the analysis due to technical failures during data acquisition, resulting in a final sample size of 13 participants. Normality of data distributions was assessed using the Lilliefors test. As the normality assumption was violated, nonparametric statistical methods were employed. An apriori power analysis was performed using G*Power 3 software, assuming a repeated‐measures design with five conditions per participant, an effect size of 0.40, a sample size of 13 participants, and a desired statistical power of 0.95 [25, 26]. Statistical significance was set at *p* < 0.05. Accordingly, results are reported as median {interquartile range, IQR}.

To evaluate whether exposure to feedback influenced performance in subsequent no‐feedback trials, a Wilcoxon rank‐sum test was first conducted to compare the two no‐feedback conditions (NoFB1 vs. NoFB2). As no significant differences were observed between these conditions (see next section), they were combined into a single NoFB group for subsequent analyses. Differences across all feedback conditions, including NoFB, were assessed using Friedman's test for repeated measures. Post hoc pairwise comparisons were performed using Wilcoxon signed‐rank tests with Holm correction to control for multiple comparisons. Effect sizes were calculated for all pairwise comparisons using the rank‐biserial correlation (*r*), and 95% confidence intervals (CI) were computed for the estimated paired differences.

## 3 Results

**FIGURE 2:**
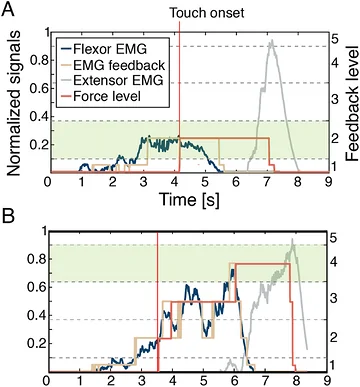
(A) Example of a successful trial with the use of osseoperceptive EMG feedback for predictive control. The participant modulated wrist flexor EMG activity to reach and maintain the second target level (green area) until the prosthesis fully closed. (B) Example of a successful trial with correction. The participant failed to reach the fourth target level before the prosthesis closed, resulting in a lower generated force. Nevertheless, the participant then used the feedback to continue increasing the myoelectric signal to the correct level, and in response the prosthesis eventually generated the desired force. Each plot shows the flexor and extensor EMG signals, the corresponding grasping force level, and the discrete feedback signals provided to the participant through vibrotactile stimulation (see Figure 1C,D). At the end of each trial, the participant generated extensor signals to open the hand.

Figure 2 illustrates two representative successful trials where a participant used osseoperceptive feedback for predictive force control. In the first case (Figure 2A), the participant increased and maintained the muscle activation (blue signal) at the correct level (level 2) during prosthesis closing, and in response, the prosthesis generated the desired force (level 2) immediately upon contact, demonstrating the so‐called routine grasping approach. The second example (Figure 2B) shows that the participant was not quick enough to achieve the desired myoelectric level during prosthesis closing, and hence the initially generated force was below the desired level. Nevertheless, the participant then continued increasing their muscle contraction, guided by the feedback, until reaching the correct level. The prosthesis responded by abrupt increases in the grasping force, successfully reaching the correct level close to the trial end. In the present analysis, all successful trials were pooled regardless of the strategies used to reach the target force (e.g., before contact or after contact).

### 3.1 Success Rate Across Feedback Conditions

**FIGURE 3:**
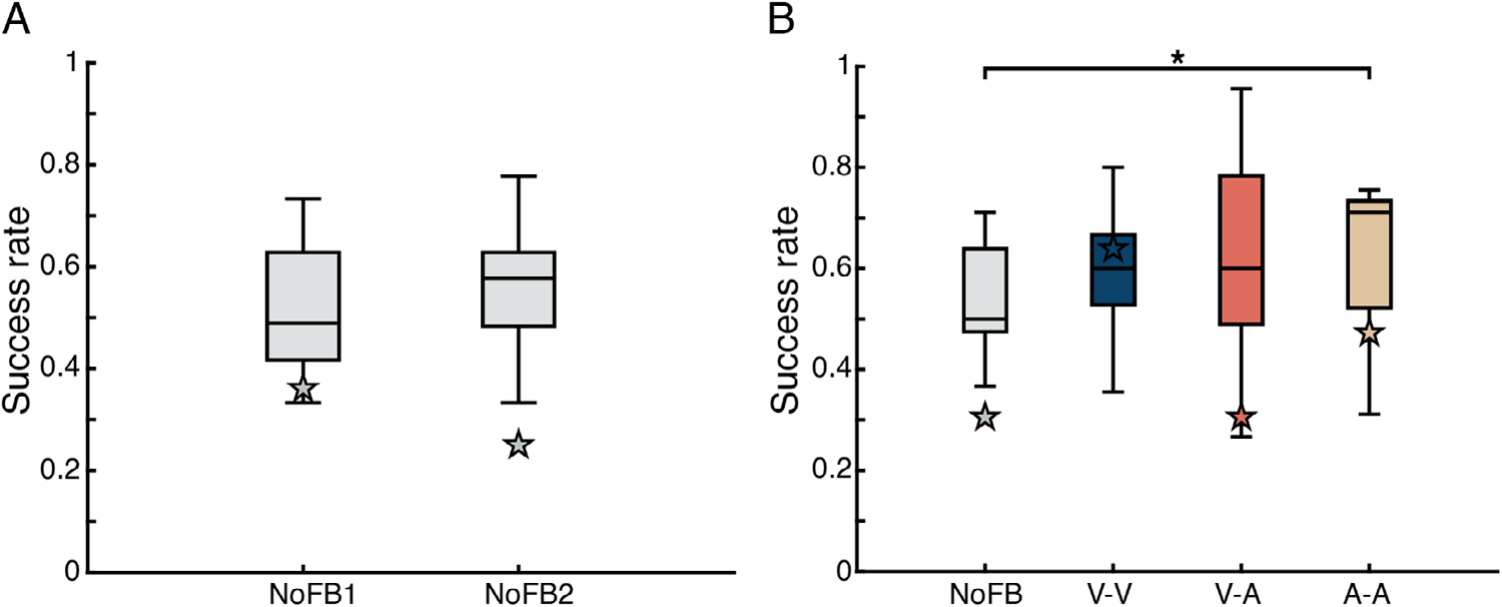
Overall task success rates across feedback conditions. (A) Success rates for the two NoFB conditions. (B) Success rates for NoFB (NoFB1 and NoFB2 together) and the three osseoperceptive feedback modes—vibrotactile (V–V), auditory (A–A), and combined (V–A). Boxplots show median (central line) and interquartile range (boxes), with whiskers representing minimum and maximum values. Asterisks indicate statistically significant differences (*p* < 0.05). The star marker represents the performance of the transradial amputee participant.

The overall success rates (median {interquartile range}) for non‐disabled participants in the two no‐feedback conditions were 48% {21%} and 58% {14.4%}, respectively (Figure 3A). The two no‐feedback conditions did not differ significantly (*p* > 0.05; effect size = −0.47, 95% CI [−0.86, 0.01]) and therefore were combined into a single NoFB condition for subsequent analyses.

A significant overall effect of feedback condition was observed (Figure 3B), with a small‐to‐moderate effect size (*p* < 0.05, effect size = 0.16, 95% CI [0.07, 0.43]). Across conditions, feedback resulted in higher median success rates and generally narrower distributions compared with NoFB. Pair‐wise comparisons indicated that the A–A condition produced the highest success rate (71.1% {21.1%}) and was significantly higher than the NoFB condition (50% {16.4%}), corresponding to a large effect (*p* < 0.05, effect size = −0.66, 95% CI [−0.50, −0.10]). In contrast, the differences between NoFB and the remaining feedback conditions did not reach statistical significance. The V–V condition resulted in a success rate of 60% {14%} (*p* > 0.05, effect size = −0.29, 95% CI [−0.71, 0.29]), while the V–A condition resulted in a success rate of 60% {29%} (*p* > 0.05, effect size = −0.45, 95% CI [−0.80, 0.07]).

Similar trend between feedback and no feedback was observed in the participant with transradial amputation, however, the highest success rate was obtained in the V–V condition (63.9%), followed by A–A (47.2%), V–A (31%), and NoFB (31%). Interestingly, the amputee's success rate decreased between the first and second no‐feedback condition (36.1% to 25%).

### 3.2 Effect of Target Level Force

**FIGURE 4:**
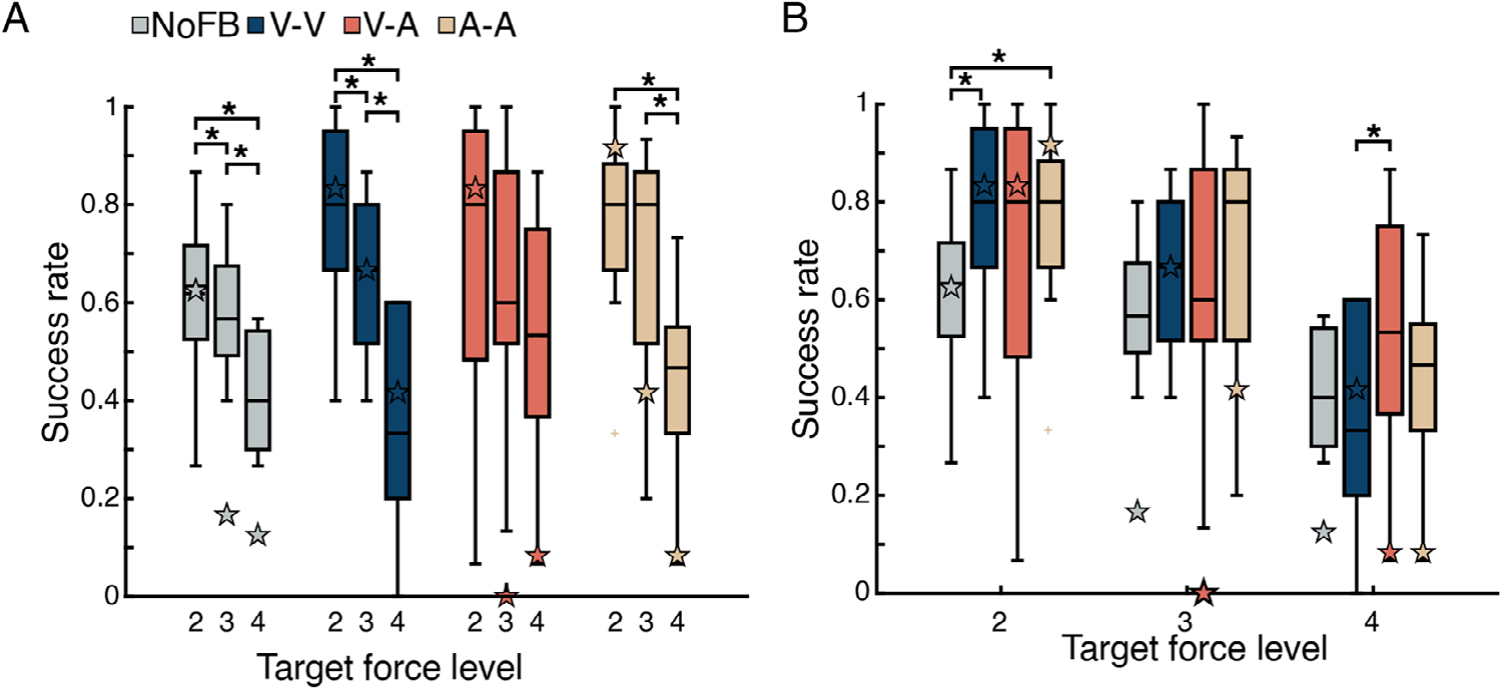
The success rate for each target force level and feedback condition. The results are grouped to analyze participants’ performance for different force levels in the same condition (A), and to compare their performance in feedback conditions for each specific target level (B). The central line within each box indicates the median, and the whiskers represent the IQR of healthy participants. Asterisks indicate statistically significant differences between conditions (*p* < 0.05). The star marker denotes the result for the amputee participant.

Figure 4A shows success rates stratified by target force level and feedback condition. For non‐disabled participants, success rate generally decreased with increasing force level. Significant reductions were observed at the highest force level (level 4) in several conditions, including A–A (*p* < 0.05, effect size = 0.44, 95% CI [0.14, 0.82]), V–V (*p* < 0.05, effect size = 0.76, 95% CI [0.58, 0.93]), and NoFB (*p* < 0.05, effect size = 0.47, 95% CI [0.23, 0.82]). No significant differences across force levels were observed in the V–A condition (*p* > 0.05, effect size = 0.19, 95% CI [0.01, 0.69]). The amputee participant exhibited a similar trend in most conditions, with even lower success rates at higher force levels.

Comparisons across feedback conditions for each force level (Figure 4B) show the overall trend of higher medians and success rate distributions in feedback conditions, especially in levels 2 and 3 for all feedback approaches and level 4 for V‐A. The significant differences in performance were registered at force level 2, where A–A and V–V outperformed NoFB (*p* < 0.05, effect size = 0.27, 95% CI [0.03, 0.72]), and at force level 4, where V–A outperformed V–V (*p* < 0.05, effect size = −0.67, 95% CI [−0.88, 0.28]). For the transradial amputee participant, the highest success rates were observed in the V–V condition at force levels 3 and 4, and in the A–A condition at force level 2. In contrast, performance in the V–A condition resulted in a 0% success rate at force level 3.

### 3.3 Task Completion Time

**FIGURE 5:**
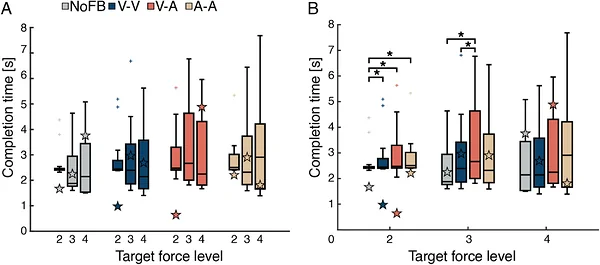
The completion time for each target force level and feedback conditions. The results are grouped to analyze participants’ performance for different force levels in the same condition (A), and to compare their performance in feedback conditions for each specific target level (B). The central line within each box indicates the median, and the whiskers represent the IQR of healthy participants. Asterisks indicate statistically significant differences between conditions (*p* < 0.05). The star marker denotes the result for the amputee participant.

In able‐bodied participants, the overall task completion times did not differ significantly across feedback conditions (Figure 5A, *p* > 0.05, effect size = 0.07, 95% CI [0.01, 0.45]). Median completion times for non‐disabled participants ranged from 1.98 s {1.04 s} to 2.58 s {1.68 s} across conditions.

When analyzed by force level (Figure 5B), significant differences emerged, mostly reflecting an increase in the task time when the feedback was provided. At force level 2, the participants were faster in NoFB than in all feedback conditions (*p* < 0.05, effect size = 0.24, 95% CI [0.15, 0.49]). At force level 3, NoFB resulted in faster completion times than V–A (*p* < 0.05, effect size = −0.59, 95% CI [−0.88, −0.13]). In addition, when using V–A feedback, the participants were slower compared to V–V (*p* < 0.05, effect size = −0.67, 95% CI [−0.86, −0.26]). At force level 4, no significant differences were found for completion time (*p* > 0.05, effect size = 0.06, 95% CI [0.01, 0.39]).

For the amputee participant, there was no consistent trend, as she was sometimes slower in NoFB conditions. The fastest completion times were obtained in V–A at force level 2, NoFB at force level 3, and A–A at force level 4. The task time with V–V, which was the best‐performing feedback for this participant, was mostly in between those achieved in the other conditions.

### 3.4 Confusion Matrix Analysis

**FIGURE 6:**
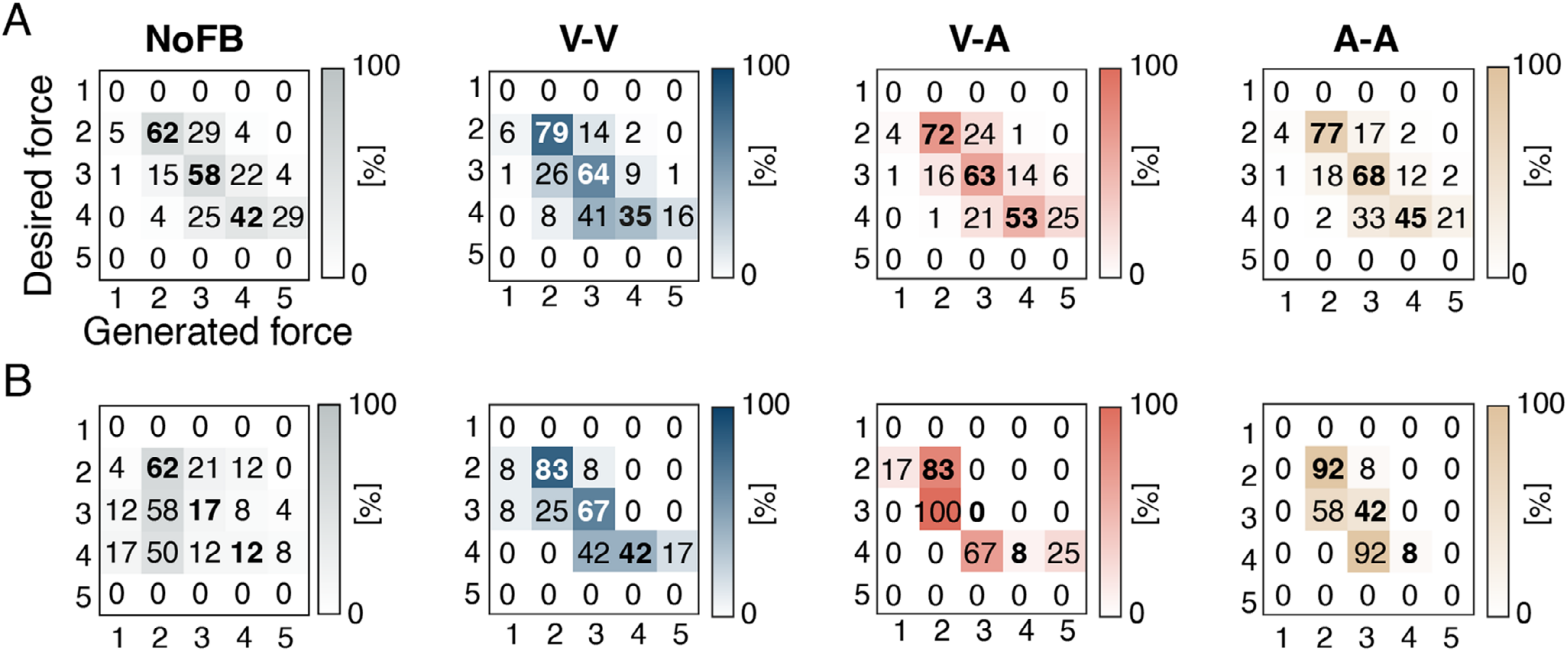
Confusion matrices of desired versus generated force levels across feedback conditions. (A) Results averaged across all non‐disabled participants. (B) Results for the amputee participant. Each matrix shows the correspondence between the desired force level (rows) and the force level produced by the participant (columns). Values are expressed as percentages per target class, such that each row sums to 100%.

Confusion matrices comparing desired and generated force levels are shown in Figure 6. For non‐disabled participants, most errors occurred at the highest force level (level 4). Confusion matrices for V–A and A–A have sharper diagonals compared to the other conditions, implying better overall accuracy. Importantly, in all conditions, when the participants failed to reach the desired force, the error was in most cases confided to one level above or below the target.

For the amputee participant, high confusion rates were observed between adjacent force levels in several conditions, particularly in V–A and A–A, and NoFB conditions. For instance, the participant missed the target force in most cases for force levels 2 and 3 in V–A and NoFB, and for force level 3 in A–A. The highest accuracies across all force levels were achieved in the V–V condition. Furthermore, the confusion rates for force level 1 have decreased under feedback conditions, in comparison to NoFB.

### 3.5 Subjective Evaluation

**FIGURE 7:**
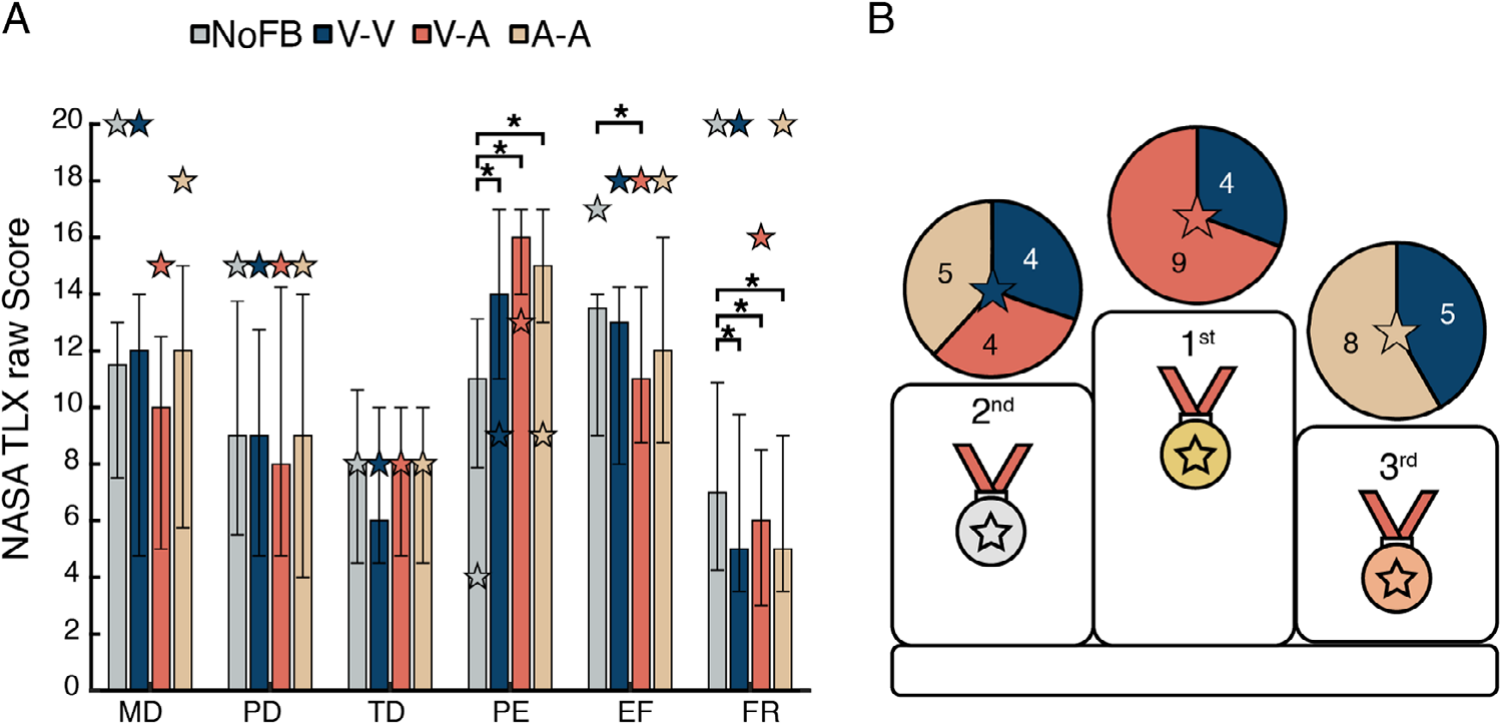
Subjective ratings and feedback preference. (A) NASA‐TLX scores across workload categories (mental—MD, physical—PD, and temporal demand—TD; performance—PE; effort—EF; and frustration—FR). Bars represent median values, and error bars indicate the interquartile range (IQR) across non‐disabled participants. (B) Ranking of preferred feedback schemes across all participants. Asterisks indicate statistically significant differences between conditions (*p* < 0.05). The star marker denotes the result for the amputee participant.

NASA‐TLX results are summarized in Figure 7A. For non‐disabled participants, perceived performance was significantly lower in NoFB compared to all feedback conditions (*p* < 0.05, effect size = 0.43, 95% CI [0.26, 0.71]). Effort ratings were lower in V–A than in NoFB (*p* < 0.05, effect size = −0.14, 95% CI [−0.54, −0.19]). Frustration was highest in NoFB and significantly reduced in all feedback conditions (*p* < 0.05, effect size = 0.22, 95% CI [0.10, 0.51]). The amputee participant reported higher perceived performance and lower frustration in feedback conditions, particularly V–A.

Preference rankings indicated that V–A was the most preferred feedback modality (first choice for 9 participants), followed by V–V. A–A was most frequently ranked as the least preferred option (Figure 7B). The amputee participant ranked V–A, V–V, and A–A as first, second, and third preference, respectively.

## 4 Discussion

This study investigated the feasibility of exploiting osseoperception, via non‐invasive bone conduction, to deliver supplementary EMG feedback for improving grasping force control when using a robotic hand prosthesis. The results demonstrated that auditory‐only (A–A) osseoperceptive feedback significantly improved force‐control accuracy compared to the no‐feedback condition, whereas vibrotactile‐only (V–V) and combined vibrotactile–auditory (V–A) feedback showed positive but non‐significant trends. In contrast, the combined vibrotactile‐auditory (V–A) modality was most preferred subjectively. Together, these findings demonstrate the feasibility of osseoperceptive EMG feedback as a non‐invasive approach for closing the prosthetic control loop. From a practical viewpoint, the present study shows that EMG feedback can be provided using a single bone conduction stimulator (contrary to previous works in the literature [3, 16], where such feedback was implemented using the arrays of conventional vibromotors).

### 4.1 Osseoperceptive EMG Feedback can Enhance Grasp Force Control

By providing continuous information about the user's own myoelectric activation, osseoperceptive EMG feedback enabled participants to regulate muscle activation during prosthesis closing, which likely contributed to the improved performance observed relative to no‐feedback conditions. Unlike force feedback derived from prosthetic sensors—which becomes informative only after contact—EMG feedback is available throughout prosthesis closing, supporting predictive regulation of grasp force, as previously reported for vibrotactile EMG feedback systems [3, 17]. The benefits of EMG feedback were demonstrated in previous studies, where this feedback was provided using conventional electro‐ and vibro‐tactile feedback interfaces. These studies showed that EMG feedback improved performance with respect to no feedback [27], as well as that it outperformed force feedback during normal grasping [28] and when the control signal was exposed to unexpected disturbances [20]. The present study confirms the finding that EMG feedback is beneficial and also shows the advantages of implementing such feedback using multimodal osseoperception. In the present system, the estimated end‐to‐end latency was approximately 120 ms, based on the nominal delays of the individual hardware components. Importantly, none of the participants remarked that they felt any perceptible delay between their muscle activation and perceived feedback. In addition, the predictive nature of EMG biofeedback reduces the need for rapid post‐contact corrections, potentially mitigating the impact of delays compared to, for instance, force‐based feedback strategies [20]; however, this is yet to be experimentally tested.

Under no‐feedback conditions participants relied solely on incidental visual and proprioceptive cues, which are indirect and cognitively demanding, resulting in higher error rates despite performance remaining above chance level (Figure 3). This indicates that while natural muscle proprioception can support rudimentary force regulation, it is insufficient for precise modulation across multiple force levels. The observed benefits of osseoperceptive EMG feedback are consistent with sensorimotor control frameworks in which feedforward predictions are refined through sensory information to reduce uncertainty and improve motor performance [29, 30].

Among the tested modalities, auditory osseoperceptive feedback (A–A) yielded the highest overall success rate. Auditory cues may offer higher perceptual resolution and temporal saliency than vibrotactile cues, even though they are non‐somatotopic. Previous studies have similarly reported the advantages of auditory feedback for grip force regulation, particularly in threshold‐based tasks involving fragile objects [31]. The advantage of osseoperception is that it can provide high‐fidelity EMG feedback and yet still ensure a self‐contained system, contrary to alternative approaches (e.g., using headphones to provide auditory stimulation [32]).

Despite this, vibrotactile‐only feedback (V–V) also demonstrated robust performance at lower force levels and was particularly effective for the amputee participant. This effect may be attributed to the intuitive mapping between vibration intensity and force magnitude. When delivered through bone conduction, vibrations at different amplitudes may evoke distinct sensations through osseous structures, potentially engaging a broader set of mechanoreceptive pathways in the periosteum than skin‐based vibrotactile stimulation using conventional motors [24, 33].

The combined modality (V–A) did not outperform unimodal feedback in objective metrics, yet it was most preferred by participants and produced relatively low confusion rates (Figures 6 and 7). This suggests that multimodal feedback may enhance perceived richness and confidence, even if it does not immediately translate into a statistically significant increase in accuracy. Interestingly, subjective preference did not directly mirror objective task performance. Although the A–A condition achieved the highest overall success rate, this was the less preferred condition. This dissociation between performance and preference highlights a critical design consideration for closed‐loop prosthetic systems: user acceptance may depend more on perceived comfort, intuitiveness, and reduced confusion than on maximal task accuracy alone [34]. Similar discrepancies between objective performance and subjective preference have been reported in multimodal feedback studies [11]. One possible explanation is that combining auditory and vibrotactile cues increases the salience and redundancy of the feedback signal, making it easier for users to detect and interpret changes in force, even if this redundancy does not improve force discrimination. In addition, participants may have weighed the more familiar or salient modality when uncertain, while still benefiting from the presence of a secondary cue as a confirmatory signal (transition between vibrotactile and auditory signals). This finding supports the notion that intuitiveness and discriminability of artificial sensory feedback are not solely reflected by task success metrics but can be better captured by confusion patterns across stimulus levels [11].

Importantly, although all feedback modalities resulted in higher median success rates compared with no feedback, the statistically supported improvement was observed only for the auditory‐only (A–A) condition (Figure 3). The remaining modalities showed positive trends with smaller effect sizes, but their confidence intervals overlapped with zero, indicating greater variability across participants and preventing definitive conclusions regarding their effectiveness.

### 4.2 Effect of Target Force Level on Feedback Effectiveness

As expected, success rates decreased with increasing target force levels with the middle force levels being the most frequently confused. This pattern is consistent with previous vibrotactile feedback studies, where intermediate and high force levels are more difficult to generate consistently [3].

The reduced performance observed at higher force levels may reflect limitations of the discrete encoding scheme, inter‐subject variability in osseoperceptive sensitivity, and increased motor noise at higher EMG activations. The fact that performance degradation at higher target force levels was not uniform across feedback modalities could indicate that this was due to the participants failing to discriminate the feedback encodings. While mid‐to‐high force levels (particularly level 4) were most frequently confused across conditions, the extent of this confusion varied with the encoding scheme, indicating that certain modalities provided more reliable perceptual spacing between force levels (e.g., V–A and A–A). The amputee participant might have exhibited similar difficulties, particularly under V–A and A–A conditions, and this could explain the discrepancy between their results and those of able‐bodied participants. In this study, a common osseoperceptive encoding structure—defined by the number of feedback levels, their ordinal mapping to EMG activation, and the associated sensory modality—was applied across participants to evaluate the feasibility of a generalized feedback strategy (Figure 1E) [24]. Prior to the experimental tasks, stimulation parameters were individually adjusted to ensure that each participant could reliably perceive and discriminate all five stimulation levels within each feedback condition, thereby confirming perceptual access to the intended mapping.

Nevertheless, the partial mitigation of performance degradation at higher force levels observed in some modalities suggests that fixed encoding structures, even when perceptually discriminable in isolation, may be more difficult to interpret during online control. Despite successful discrimination during the initial verification phase, participants may have experienced increased uncertainty when integrating the feedback in real time with motor execution. Together with the observed inter‐individual variability in response to feedback, these findings indicate that osseoperceptive feedback may benefit from individualized or dynamically adapted encoding parameters. Furthermore, exploring a broader range of stimulation frequencies or frequency‐modulated feedback within the osseoperceptive spectrum (200–500 Hz) may enhance intuitiveness and perceptual spacing, offering promising avenues for user‐specific optimization.

### 4.3 Speed‐Accuracy Trade‐Off and Cognitive Workload

The NASA‐TLX results further suggest that osseoperceptive feedback reduced perceived workload relative to the no‐feedback condition, despite longer task execution times. This pattern indicates that participants were willing to trade speed for improved confidence and control when meaningful sensory information was available. Such a trade‐off [35] is consistent with previous studies showing that artificial feedback can increase task duration while simultaneously reducing mental demand and perceived effort [3, 22]. Furthermore, although feedback increased task duration, it also improved perceived control, suggesting that users may prioritize accuracy and stability over speed. However, these outcomes should be interpreted cautiously, as both cognitive load and task duration depend on task complexity, user experience with myoelectric control, and familiarization with the feedback modality [15, 18].

### 4.4 No Evidence for Short‐Term Training Effect of Osseoperceptive Feedback

Previous work has shown that artificial force feedback can serve as an effective training tool, enabling users to develop internal models that support feedforward control even after feedback removal [16, 36]. In the present study, however, no significant improvement was observed between the initial and final no‐feedback conditions.

This discrepancy may be explained by the continuous availability of EMG feedback during muscle activation, which may have encouraged participants to rely on the artificial feedback rather than on intrinsic proprioceptive cues [19]. Additionally, the short duration of exposure may have limited learning transfer, but both of these points need to be tested in the future longitudinal study.

### 4.5 Comparison with Existing Feedback Approaches

The success rates achieved with osseoperceptive feedback are comparable to those reported for vibrotactile EMG feedback in the literature [3]. While direct comparisons are limited by differences in task design and force resolution, osseoperceptive feedback offers practical advantages, including reduced hardware complexity and independence from skin condition or available surface area.

Unlike many auditory feedback approaches that rely on external headphones [31, 37], osseoperception enables auditory sensations via bone conduction without occluding the ears, enhancing applicability to more realistic scenarios for prosthetic users. Importantly, the present study extends prior binary osseoperceptive feedback paradigms to a multi‐level force regulation task, which is closer to real‐world manipulation demands [22].

While the present study focused on grip‐force regulation during functional grasping tasks, the results suggest that osseoperceptive feedback could support broader closed‐loop prosthetic control strategies [38, 39, 40]. In particular, the ability to convey vibrotactile and auditory‐like cues through a single bone‐conducted actuator highlights the versatility of this approach for delivering supplementary sensory information in a non‐invasive manner. Moreover, compatibility with commercial prostheses and minimal hardware requirements make osseoperceptive feedback a promising complement to existing non‐invasive sensory substitution methods [6]. In this context, direct comparisons—or complementary integration—of osseoperceptive and other feedback strategies (e.g., electrotactile, vibrotactile, etc) may also provide valuable insights into how different modalities can be optimally combined to enhance prosthetic control [15].

### 4.6 Insights from the Amputee Participant

This case study provides preliminary evidence that osseoperceptive EMG feedback can be implemented during functional prosthetic control in a transradial amputee. The participant achieved the highest performance with vibrotactile feedback (V–V) with an approx. 30% increase in performance, contrary to the results from non‐disabled participants, where A–A led to the highest success rate. This discrepancy may reflect individual differences in perceptual sensitivity to the tested modalities (tactile and auditory), which may be also related to age difference. Additionally, the fact that the amputee participant did not use the socket might have allowed better focus on the “pure” vibrotactile sensations [18, 34]. Although these observations demonstrate technical feasibility, no clinical conclusions can be drawn from a single participant. Larger studies involving prosthesis users will be necessary to determine the clinical applicability of the proposed approach.

### 4.7 Limitations and Future Work

Several limitations should be acknowledged. First, although the study included thirteen non‐disabled participants and one transradial amputee, the main evaluation was performed using a bypass prosthesis operated by non‐disabled participants. This experimental framework has been widely adopted in prosthetic research to investigate control strategies and sensory feedback mechanisms while reducing confounding factors associated with socket fit and residual limb variability [41]. However, it does not fully reproduce prosthesis use in individuals with amputation, as participants retained intact proprioceptive pathways and interacted with an externally mounted prosthesis with wrist immobilization under controlled laboratory conditions. Therefore, the ecological validity of the findings is limited, and future studies should evaluate osseoperceptive feedback in larger cohorts of prosthesis users using wearable prosthetic systems during realistic daily‐life tasks. Second, the bone‐conduction transducer was not integrated into a prosthetic socket, limiting evaluation of how osseoperceptive feedback would function during a real‐life prosthesis scenario. Third, the study involved a single‐session assessment, preventing analysis of long‐term learning effects and adaptation to the feedback [16]. Despite these constraints, the results demonstrate the feasibility of osseoperceptive EMG feedback for supporting grasp‐force control and indicate its potential as a non‐invasive approach for closing the loop in upper limb prostheses. Future studies will address these limitations by including larger amputee cohorts, integrating the bone transducer within wearable prosthetic systems, and evaluating performance over extended training periods in more realistic daily‐living tasks.

## Funding

The work of Leonardo Cappello was supported by the European Union (ERC, MUSE) under Grant 101116249. Views and opinions expressed are however those of the author(s) only and do not necessarily reflect those of the European Union or the European Research Council. Neither the European Union nor the granting authority can be held responsible for them. The work of Strahinja Dosen was supported by the project DexterHand funded by INAIL (Istituto Nazionale per l'Assicurazione contro gli Infortuni sul Lavoro) under Grant Agreement PR23‐PAS‐P1 – DexterHand.

## Ethics Statement

All participants provided written informed consent prior to participation. The experimental protocol was approved by the Ethics Committee of the North Denmark Region (Approval No. N‐20250021) and was conducted in accordance with the Declaration of Helsinki.

## Conflicts of Interest

The authors declare no conflicts of interest.

## Data Availability

The datasets generated and/or analyzed during the current study are available from the corresponding author on reasonable request.
